# Palpebral Fissure Response to Phenylephrine Indicates Autonomic Dysfunction in Patients With Type 1 Diabetes and Polyneuropathy

**DOI:** 10.1167/iovs.63.9.21

**Published:** 2022-08-18

**Authors:** Thomas Arendt Nielsen, Carl Uggerhøj Andersen, Henrik Vorum, Sam Riahi, Rok Sega, Asbjørn Mohr Drewes, Jesper Karmisholt, Poul Erik Jakobsen, Birgitte Brock, Christina Brock

**Affiliations:** 1 Department of Ophthalmology, Aalborg University Hospital, Aalborg, Denmark; 2 Department of Clinical Medicine, Aalborg University, Aalborg, Denmark; 3 Mech-Sense, Department of Gastroenterology and Hepatology, Aalborg University Hospital, Denmark; 4 Department of Cardiology, Aalborg University Hospital, Denmark; 5 Department of Ophthalmology, University Medical Centre Ljubljana, Ljubljana, Slovenia; 6 Department of Endocrinology, Aalborg University Hospital, Aalborg, Denmark; 7 Steno Diabetes Center North Denmark, Aalborg, Denmark; 8 Steno Diabetes Center Copenhagen, Gentofte, Denmark; 9 Department of Clinical Biochemistry, Aarhus University Hospital, Aarhus, Denmark

**Keywords:** diabetes, heart rate variability, autonomic neuropathy, palpebral fissure, diabetic retinopathy

## Abstract

**Purpose:**

The superior and inferior tarsal muscles are sympathetically innervated smooth muscles. Long-term diabetes often leads to microvascular complications, such as, retinopathy and autonomic neuropathy. We hypothesized that diabetes induces (1) sympathetic paresis in the superior and inferior tarsal muscles and that this measure is associated with (2) the severity of diabetic retinopathy, (3) the duration of diabetes, and (4) autonomic function. In addition, association between the severity of retinopathy and autonomic function was investigated.

**Methods:**

Forty-eight participants with long-term type 1 diabetes and confirmed distal symmetrical polyneuropathy were included. Palpebral fissure heights were measured bilaterally in response to topically applied 10% phenylephrine to the right eye. The presence of proliferative diabetic retinopathy (PDR) or nonproliferative diabetic retinopathy and disease duration were denoted. Time and frequency derived heart rate variability parameters obtained from 24-hour continuous electrocardiography were recorded.

**Results:**

The difference in palpebral fissure heights between phenylephrine treated and untreated eyes (∆PFH) was 1.02 mm ± 0.29 (*P* = 0.001). The ∆PFH was significantly lower in the PDR group (0.41 mm ± 0.43 vs. 1.27 mm ± 1.0), F(1,35) = 5.26, *P* = 0.011. The ∆PFH was lower with increasing diabetes duration, r(37) = −0.612, *P* = 0.000. Further, the ∆PFH was lower with diminished autonomic function assessed as total frequency power in electrocardiogram (r = 0.417, *P* = 0.014), and sympathetic measures of very low (r = 0.437, *P* = 0.010) and low frequency power (r = 0.384, *P* = 0.025).

**Conclusions:**

The ∆PFH is a simple ambulatory sympathetic measure, which was associated with the presence of PDR, disease duration, and autonomic function. Consequently, ∆PFH could potentially be an inexpensive and sensitive clinical indicator of autonomic dysfunction.

Sensory-motor and autonomic neuropathies are well-described microvascular complications to long-term diabetes. Major clinical manifestations of diabetic autonomic neuropathy (DAN) include impairment of the nervous supply to the heart, gastrointestinal tract, and urogenital systems, as well as impaired neurovascular function.^1^ Routine clinical examinations do not include a thorough investigation of the autonomic nervous system, even though the presence of DAN is the strongest predictor of all-cause mortality, arrhythmias, and sudden death.^2^

The superior and inferior tarsal muscles are sympathetically innervated smooth muscles involved in sustaining eyelid retraction. The innervation originates from nerve fibers in the superior cervical ganglion. Consequently, topical application of phenylephrine, a sympathomimetic drug, causes an increase in palpebral fissure height (PFH) in healthy subjects of 1.2 to 1.6 mm accordingly.^3^^–^^5^ In diabetes, however, the PFH is significantly decreased compared with healthy controls. On average, people with diabetes who depend on insulin had a PFH of 8.3 mm, whereas healthy controls had a PFH of 9.9.^6^ This decrease is associated with disease duration (in insulin-dependent diabetes) and with the severity of diabetic retinopathy (DR).^6^ Thus, PFH may reveal a proxy of the autonomic sympathetic function and potentially serve as a cheap, sensitive clinical indicator of the sympathetic drive.

Bilaterally diminished PFH is indicative of sympathetic paresis. The interpretation of this measure is more straightforward than evaluating pupillary responses, because the pupil receives dual sympathetic and parasympathetic innervation. Nevertheless, pupillary denervation hypersensitivity to pilocarpine has shown to be an indicator of early DAN,^7^ reflected clinically as, such as, smaller pupils (miosis) or decreased light reflex amplitudes.^8^^,^^9^

Topical application of the alpha-adrenergic agonist phenylephrine in one eye provides the possibility of evaluating the pupillary sensitivity and PFH difference between the two eyes (∆PFH), which could serve as an indicator of sympathetic function. For example, miosis is associated with cardiac vagal dysfunction and somatic sensory loss.^10^^,^^11^ Furthermore, it has been shown that pupillary autonomic neuropathy was associated with the severity of DR and cardiac autonomic neuropathy (CAN)^12^; however, measures of PFH need to be validated against classical heart rate variability measures.

Another common microvascular complication of diabetes mellitus is DR. Because it shares the same pathogenesis, it is not surprising that several studies have shown associations between severity of retinopathy and autonomic function.^13^^–^^16^ Currently, cardiac autonomic reflex tests, including the Ewing battery,^17^ are the golden standard to diagnose CAN, however, 24-hour electrocardiograms derived time- and frequency-domain heart rate variability parameters,^18^^,^^19^ are often used to evaluate the sympathovagal balance.^20^ These tests are, however, comprehensive, resource demanding, time consuming, and require preparation and patient compliance. Therefore, alternative measures of autonomic function are warranted.

Consequently, we hypothesized that long-term diabetes induces sympathetic paresis in the peripheral sympathetic nerve fibers innervating the superior and inferior tarsal muscles. Thus, the aim of this study was to (1) quantify the sympathetic paresis by assessing eye-to-eye differences in PFH (∆PFH) after topical application of 10% phenylephrine and to associate this measure with (2) the severity of DR, (3) disease duration, and (4) autonomic function in patients with long-term diabetes. In addition, we investigated (5) the association between autonomic function and the severity of DR.

## Methods

### Study Population

These data are secondary analyses of baseline assessments in a larger randomized clinical trial, registration number (EUDRA CT, ref 2013–004375-12; clinicaltrials.gov, ref NCT02138045).^21^ Before entry, all participants provided written informed consent. The North Denmark Region (N-20130077) granted ethical approval, and the study was performed in accordance with the International Council for Harmonization guideline for Good Clinical Practice and the Declaration of Helsinki. Forty-eight participants (38 male; median age, 50 years; interquartile range, 33–71 years) with long-term type 1 diabetes and severe concomitant distal symmetric polyneuropathy diagnosed based on the Toronto criteria were included.^20^ Additional inclusion criteria were stable diabetes treatment (long/fast-acting insulin or insulin pump) for a minimum of 3 months before entering the study, age over 18 years, and a body mass index of greater than 22. Exclusion criteria were type 2 diabetes, neurological disorders other than distal symmetric polyneuropathy, psychiatric diseases, a hemoglobin A1c level of less than 7% (48 mmol/mol), and treatment for other endocrine disorders. Additional exclusion criteria were previous eyelid trauma or surgery, preexisting ptosis, or glaucoma. Eleven participants were excluded from this substudy owing to either missing data or violations of the additional exclusion criteria; hence 37 participants were included for further analysis.

### PFH

**Figure 1.**
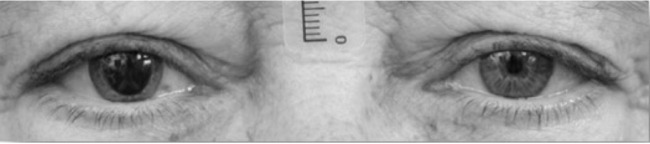
Assessment of PFH between right phenylephrine and left untreated eye.

PFH is defined as the vertical palpebral aperture height between the upper and lower eyelid margin in the pupillary plane of the eyes in the primary position of gaze. PFH was measured on both eyes by standardized frontal view photograph, 15 minutes after instillation of two drops of phenylephrine hydrochloride 10% (Bausch & Lomb Minims, Laval, Canada) into the inferior fornix of the right eye. A scale bar was placed between the subject's eyes to allow accurate life-size measurements (Fig. 1^2^). The difference in PFH (mm) between the right phenylephrine treated eye and left untreated eye was denoted as ∆PFH.

**Figure 2.**
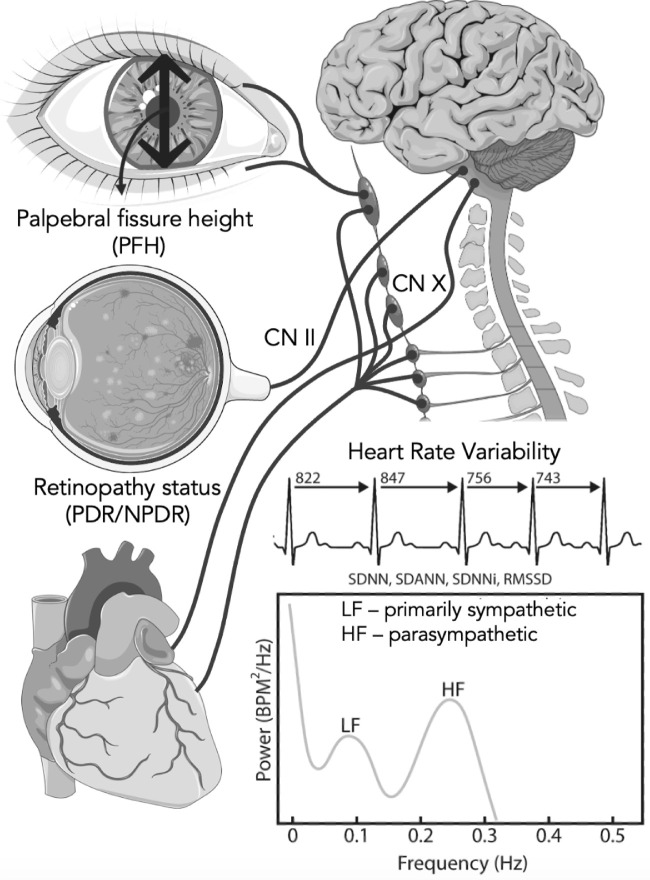
Overview of assessments. CN II, cranial nerve 2; CN X, cranial nerve 10; NPDR, nonproliferative diabetic neuropathy; RMSSD, root mean square of successive RR interval differences; SDNN, standard deviation of NN intervals; SDANN, standard deviation of all NN intervals for every 5 minutes for 24 hours; SDNNi, mean of the standard deviation of all NN intervals for every 5 minutes for 24 hours.

### DR

Participants were dichotomized into proliferative DR (PDR) and nonproliferative DR (NPDR) groups by the presence (previous or actual) or absence of neovascularization, respectively. Neovascularization is defined by the formation of new blood vessels originating from the retina and extending to the vitreoretinal interface and potentially into the vitreous. The presence of neovascularization was identified by visual inspection of fundus photos and optical coherence tomography scans. The same trained ophthalmologist and photographer performed all assessments to minimize interobserver bias. In accordance with the Danish diabetes program, individuals with diabetes are referred to a private practitioner in ophthalmology and recommended to participate in yearly checkups. If and when treatment is needed (laser, anti-VEGF, surgery, etc.), patients are referred to a hospital where these measurements were conducted. Therefore, patients who have made use of this recommendation are diagnosed and followed systematically in a secondary center, and the diagnosis was confirmed at the hospital. For patients who have not made use of this recommendation, the diagnosis was made upon examination at the hospital.

### Heart Rate Variability

The current study used the definition published by Shaffer et al.^19^ Twenty-four-hour continuous electrocardiogram was recorded (Lifecard CF; Del Mar Reynolds, Spacelabs Healthcare Inc., Snoqualmie, WA), from which the following time-domain parameters were obtained: standard deviation of NN intervals (SDNN), mean of the standard deviation of all NN intervals for every 5 minutes for 24 hours (SDNNI), standard deviation of all NN intervals for every 5 minutes for 24 hours; root mean square of successive RR interval differences (RMSSD), mean RR interval, and heart rate. Fast Fourier transformation provided the following frequency-domain heart rate variability parameters: total frequency power, very low frequency (VLF), low frequency (LF), high frequency (HF), and the LF/HR ratio. Traditionally, the VLF and LF content is believed to primarily represent the sympathetic content, whereas HF and RMSSD represent parasympathetic content.^1^^,^^22^ In addition, the LF/HF ratio reflects sympathovagal balance. See (Fig. 2) for overview of assessments.

### Statistical Analyses

Data handling and statistical computations were carried out using SPSS 25.0.0 (IBM, Armonk, NY). All data are presented as arithmetic means ± standard deviation or as medians with interquartile range depending on the distribution of the data. Data were tested for normality by visually inspecting histograms, Q–Q plots, as well as by Shapiro–Wilk testing. The average PFH for the right and left eye were calculated and compared using independent samples *t* tests. Differences in ∆PFH between the PDR and NPDR groups were tested using an independent samples *t* test. Associations between ∆PFH, disease duration, and cardiovascular autonomic function were tested using Pearson's correlation coefficient. Heart rate variability parameters were adjusted for resting heart rate at the time of testing. To test for differences in cardiovascular autonomic function and severity of DR, one-way ANOVA was used. A *P* value of 0.05 or less was considered significant.

## Results

The characteristics of the study group are shown in Table 1.

**Table 1.** Baseline Characteristics

Characteristics	Study Group
PFH	*n* = 37
PFH difference (∆PFH) (mm)	1.02 ± 0.29
Demographics	*n* = 48* *
Sex (M/F)	38/10
Age (years), range	50.0 (33–71)
Weight (kg), range	90.0 (63-132)
Body mass index (kg/m^2^)	28.5 ± 4.9
Duration of DM1 (years), range	32.2 (14–51)
Retinopathy (PDR/NPDR)	15/33
Systolic blood pressure	149.9 ± 16
Diastolic blood pressure	82.3 ± 10.9
Antihypertensive medication	33/48
Orthostatic hypotension	42/48
HRV: Frequency domain	
Total frequency power (ms^2^)	1550.5 (144–5030)
VLF (ms^2^)	1082 (117–3190)
LF (ms^2^)	378.5 (16–1474)
HF (ms^2^)	87.5 (7–624)
LF/HF	3.9 ± 2.0
HRV: time domain	
Mean RR interval (ms)	775.6 ± 105.5
SDNN (ms)	114.1 ± 34.9
SDNNi (ms)	41.4 ± 18.3
SDANN (ms)	104.7 ± 31.9
RMSSD (ms)	19.3 ± 9.6

HRV, heart rate variability; NPDR, nonproliferative diabetic retinopathy; PDR, proliferative diabetic retinopathy; RMSSD, root mean square of successive RR interval differences; SDANN, standard deviation of all NN intervals for every 5 minutes for 24 hours; SDNN, standard deviation of NN intervals; SDNNi, mean of the standard deviation of all NN intervals for every 5 minutes for 24 hours.

### PFH

**Figure 3.**
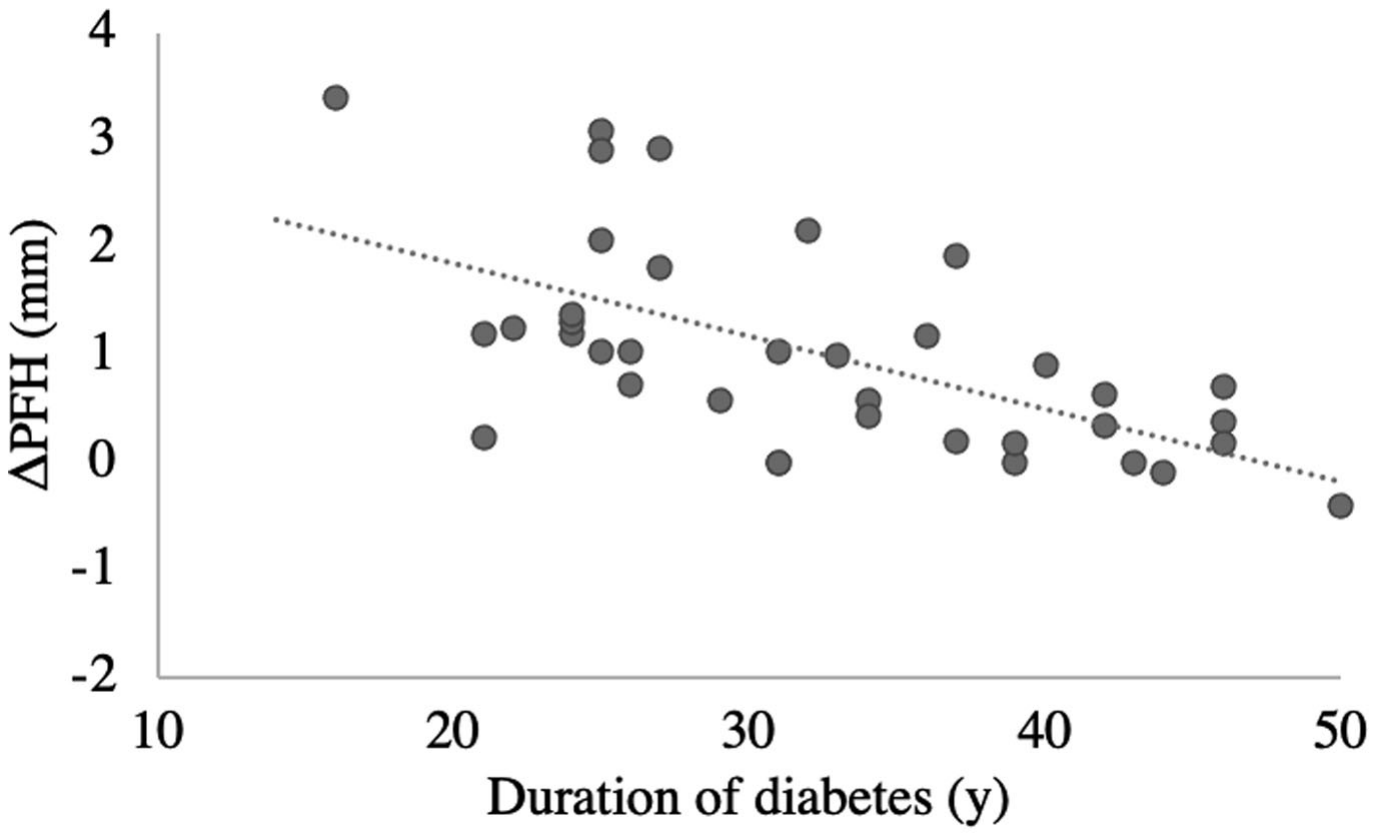
Correlations between the ∆PFH, between right phenylephrine treated and left untreated eye (mm), and duration of diabetes (years).

Instillation of 10% phenylephrine in the right eye resulted in increased eyelid retraction providing a ∆PFH of 1.02 ± 0.29 mm (treated eye 9.15 ± 1.20 mm vs. untreated eye 8.13 ± 1.30 mm; *P* = 0.001), indicating tarsal muscle response after the stimulation of adrenergic receptors. The *∆*PFH was significantly lower in the PDR group (0.41 ± 0.43 mm vs. 1.27 ± 1.0 mm), F(1,35) = 5.26, *P* = 0.011 (Table 2), indicating an association between severity of sympathetic paresis and severity of DR. Furthermore, ∆PFH was diminished with increased diabetes duration, r(37)= −0.612, *P* = 0.000 (Fig. 3). A positive association was found between the ∆PFH and the following frequency domain parameters of heart rate variability: total frequency power (r = 0.417; n = 34; *P* = 0.014), VLF (r = 0.437; n = 34; *P* = 0.010), and LF (r = 0.384; n = 34; *P* = 0.025), indicating an association between the degree of sympathetic paresis and sympathetic regulation of the heart. No associations were found in time-domain parameters. Heart rate variability measures are shown in Table 3.

**Table 2.** ∆PFH and Heart Rate Variability Measurements Between the PDR and NPDR Groups

	PDR	NPDR	*P* Value	*n*
PFH				
PFH difference (∆PFH) (mm)	0.41	1.27	0.011	11/26
HRV parameters				
Total frequency power (ms^2^)	1008.86	1914.57	0.007	14/30
VLF (ms^2^)	707.5	1252.77	0.009	14/30
LF (ms^2^)	233.57	482.3	0.017	14/30
HF (ms^2^)	51.79	153.7	0.006	14/30
SDNNi (ms)	30	46.58	0.004	14/31
RMSSD (ms)	13.36	21.94	0.004	14/31

Only significant results are included in this table.

HRV, heart rate variability; RMSSD, root mean square of successive RR interval differences; SDNNi, mean of the standard deviation of all NN intervals for every 5 minutes for 24 hours.

**Table 3.** Correlations Between ∆PFH and Cardiac Measurements

	∆PFH
HRV: time domain	
Mean RR (ms)	r = 0.021, *P* = 0.904
SDNN (ms)	r = –0.020, *P* = 0.910
SDNNi (ms)	r = 0.163, *P* = 0.351
SDANN (ms)	r = –0.092, *P* = 0.601
RMSSD (ms)	r = 0.101, *P* = 0.562
HRV: frequency domain	
Total frequency power (ms^2^)	r = 0.417, *P* = 0.014^*^
VLF (ms^2^)	r = 0.437, *P* = 0.010^*^
LF (ms^2^)	r = 0.384, *P* = 0.025^*^
HF (ms^2^)	r = 0.227, *P* = 0.197
LF/HF	r = –0.097, *P* = 0.584

* Statistically significant results.

HRV, heart rate variability; mean RR, mean R to R interval; RMSSD, root mean square of successive RR interval differences; SDNN, standard deviation of NN intervals; SDNNi, mean of the standard deviation of all NN intervals for every 5 minutes for 24 hours; SDANN, standard deviation of all NN intervals for every 5 minutes for 24 hours.

### Clinical Association Between Autonomic Nervous System Function and Severity of DR

Total frequency power, VLF, LF, HF, SDNNi, and RMSSD, were significantly lower in the PDR group in comparison with the NPDR group (*P* ≤ 0.05) (see Table 2), indicating an association between severity of the two microvascular complications, DR and autonomic function.

## Discussion

The difference in PFH, measured as ∆PFH, between topically applied phenylephrine and no treatment showed positive associations to the LF content of heart rate variability parameters, primarily representing sympathetic tone. In this specific cohort with established diabetic polyneuropathy, the ∆PFH could potentially act as an indicator of the presence or lack of sympathetic reserve capacity, which again was associated with severity of DR and diabetes duration. The method seemed sensitive to assess the function of the sympathetic fibers, because significant differences between the treated and untreated eyes were present. We believe this is the first time this simple and inexpensive sympathetic response is measured in a cohort with diabetes and verified diabetic polyneuropathy. Our findings may indicate its usefulness as a screening tool; we showed associations with established heart rate variability measures of autonomic function. However, we cannot conclude whether the method is useful in the early stages of diabetic polyneuropathy, where sympathovagal balance is less affected.

### PFH Response to Phenylephrine in DAN

DAN leads to sympathetic paresis characterized by miosis and ptosis.^6^^,^^8^ Pupillary denervation hypersensitivity to phenylephrine has been shown in diabetes^10^^,^^11^; however, to our knowledge, denervation hypersensitivity to phenylephrine has not been investigated in DAN. In the case of denervation hypersensitivity, an increased ∆PFH response would be expected. In support of this, Koc et al.^23^ investigated the effect of apraclonidine (a weak α1 receptor agonist and a potent α2 receptor agonist) in oculosympathetic paresis in 31 eyes (9 with Horner's syndrome and 22 with diabetes). They found a significant elevation of the upper lid in comparison with healthy controls,^23^ possibly owing to the upregulation and denervation hypersensitivity of the α2-receptors. Because the cohort primarily existed of patients with diabetes, this finding could indicate the existence of tarsal muscle denervation hypersensitivity to apraclonidine in DAN.

Moreover, the expression of α1D-, α2C- and β2-receptors in the superior tarsal muscle in ptotic patients have been reported in descending order^24^^,^^25^; however, α2A-receptors have also been reported as the predominant subtype.^26^ In addition, it has been shown that the eyelid elevation response to phenylephrine was inversely related to the amount of α2C-receptors in the muscle.^24^ Changes of such receptor expressions could explain the proposed denervation hypersensitivity to apraclonidine and possibly not to phenylephrine. In support of this finding, our results do not show evidence of denervation hypersensitivity to phenylephrine in DAN; ∆PFH was lower than in healthy subjects, indicating less sympathetic reserve capacity. This established sympathetic denervation is in accordance with the presence of orthostatic hypotension in 87.5% of the participants, which is also caused by sympathetic denervation, and may explain the shown associations to the severity of DR and disease duration.

### PFH

Our intention was to show whether a very simple method could make a general autonomic neuropathy probable, and thus the used method should be translated easily into daily clinical practice. Although Müller's muscle is admittedly dependent on sympathetic stimulation, it can also be influenced by other factors such as gaze direction, flexion or extension of the neck, and orbicularis oculi tone. We found a small, although highly significant difference in PFH between the treated eye and the untreated eye (∆PFH), in accordance with the measures reported by Bastiaensen et al.^6^ in a cohort of patients with insulin-dependent diabetes. The ∆PFH response in healthy particpants^3^^–^^5^ is up to 70% higher in comparison with our measures, indicating a loss of sympathetic reserve capacity caused by decreased adrenergic excitability and autonomic dysfunction**.**

Furthermore, this diminished reserve capacity was more evident in the PDR group in comparison with the NPDR group, indicating pronounced microvascular complications, and these alterations were furthermore associated with diabetes duration. To our knowledge, this study is the first time such associations have been reported.

Finally, we showed a positive association between the simple ∆PFH and advanced heart rate variability frequency measures, VLF (0.0033–0.04 Hz) and LF (0.04–0.15 Hz), which plausibly indicate the systemic sympathodenervation The understanding and impact of LF heart rate variability are, however, controversial. For example, if the LF component reflects the sympathetic cardiac tone, interventions that increase the sympathetic drive should increase the LF power. An increase in LF power has been shown by inducing myocardial ischemia^27^ and sympathetic activity by orthostatic tilt.^28^ In contrast, a decrease in LF power has been found after isoprenaline infusion,^29^ and a reflex decrease in cardiac sympathetic activity was not accompanied by changes in the LF band,^30^ indicating the presence of both parasympathetic and sympathetic content in these frequency bands.

These interpretations are further supported by the absence of associations between the ∆PFH and HF content, the LF/HF frequency ratio, and the RMSSD, which generally is accepted to reflect parasympathetic content. Still, the definitions of the heart rate variability bands have changed over the years, making study comparisons difficult.^19^^,^^31^ Consequently, the ∆PFH could be a future clinically relevant marker of sympathodenervation, which is characterized by orthostatic hypotension, cardiac arrhythmias, and sudden death.^32^

### Severity of DR and Heart Rate Variability Measures

Not surprisingly, several studies have found associations between severity of DR and autonomic dysfunction in type 1^13^^,^^14^ and type 2 diabetes,^15^^,^^16^ because they both represent long-term diabetic microvascular complications. In fact, Huang et al.^33^ have suggested that DR is the most significant predictive risk factor of CAN in patients with type 2 diabetes, and retinopathy is an independent risk marker for cardiovascular diseases, such as cardiovascular deaths,^34^ stroke, major cardiovascular events, and peripheral artery disease.^35^ Our data show that total frequency power, VLF, LF, HF, SDNNi, and RMSSD were significantly lower in the PDR group than in the NPDR group, indicating severe autonomic dysfunction. Numerous studies reported similar results, where reductions in total power,^14^ VLF,^36^ LF,^14^^,^^36^^–^^38^ HF,^14^^,^^36^^,^^38^ and LF/HF^37^^,^^38^ have shown to be associated with the severity of DR or presence of diabetes. Although there are some conflicting results, a general attenuation of heart rate variability measures seems to be evident. These findings are in accordance with our results and indicate that an attenuation of the autonomic innervation of the heart was associated with the severity of DR.

### Limitations

The major strength of this study is the ability to associate the introduced novel measure to existing validated autonomic measures of heart rate variability indicative of autonomic function. There are, however, several limitations to our study. First, no frontal photograph was taken before the phenylephrine test, allowing for assessments of pre-existing asymmetry. A previous study investigating PFH asymmetry in primary gaze did not show an affinity to one eye over the other.^39^ Because phenylephrine was always instilled into the right eye, we assume that the evoked asymmetry is equally increased or decreased and, therefore, theoretically would cancel out. Second, the external validity or generalizability to other phenotypes of diabetes such as type 1 diabetes without severe neuropathy, early diagnosed type 1 diabetes, or even type 2 diabetes remains unclear. This finding needs further clarification. Third, longstanding and pronounced severe neuropathy in this subpopulation advocating for extensive impairment of the sympathetic nerves may be caused by the long average duration of (>32 years) of diabetes, because the disease was present before the guidelines advocating for intensive glycemic control. Consequently, our findings may not reflect future patients. Fourth, the validation of PFH against sympathetic content in heart rate variability reveals the need to investigate whether a diminished ∆PFH is associated with cardiac autonomic reflex testing used for diagnosing CAN.

## Conclusions

The ∆PFH was associated with the severity of DR, disease duration, and validated heart rate variability parameters related to predominantly LF content primarily representing the sympathetic content of the sympathovagal balance in patients with type 1 diabetes and confirmed polyneuropathy. Hence, ∆PFH, a simple ambulatory measure, could potentially be a sensitive clinical indicator of autonomic dysfunction.
